# Quo Vadis Cell Growth and Division?

**DOI:** 10.3389/fcell.2016.00095

**Published:** 2016-08-30

**Authors:** Philipp Kaldis

**Affiliations:** ^1^Cell Division and Cancer Research, Institute of Molecular and Cell Biology, Agency for Science, Technology and ResearchSingapore, Singapore; ^2^Department of Biochemistry, National University of SingaporeSingapore, Singapore

**Keywords:** cell division, cell growth, cell size, cell cycle regulation, metabolism

In animal cells and models, the control of cell growth and division is essential for the maintenance of cellular homeostasis and for cell proliferation. Defects in these pathways can lead to abnormal cell proliferation and eventually development of cancer. Although cell growth and cell division are two functionally distinct processes, they are crucial for generating progeny of all cells and are strongly intertwined. Therefore, these terms are often used interchangeably, which leads to confusion. It is best then to correctly define these terms before we discuss them in detail (Figure 1).

**Figure 1:**
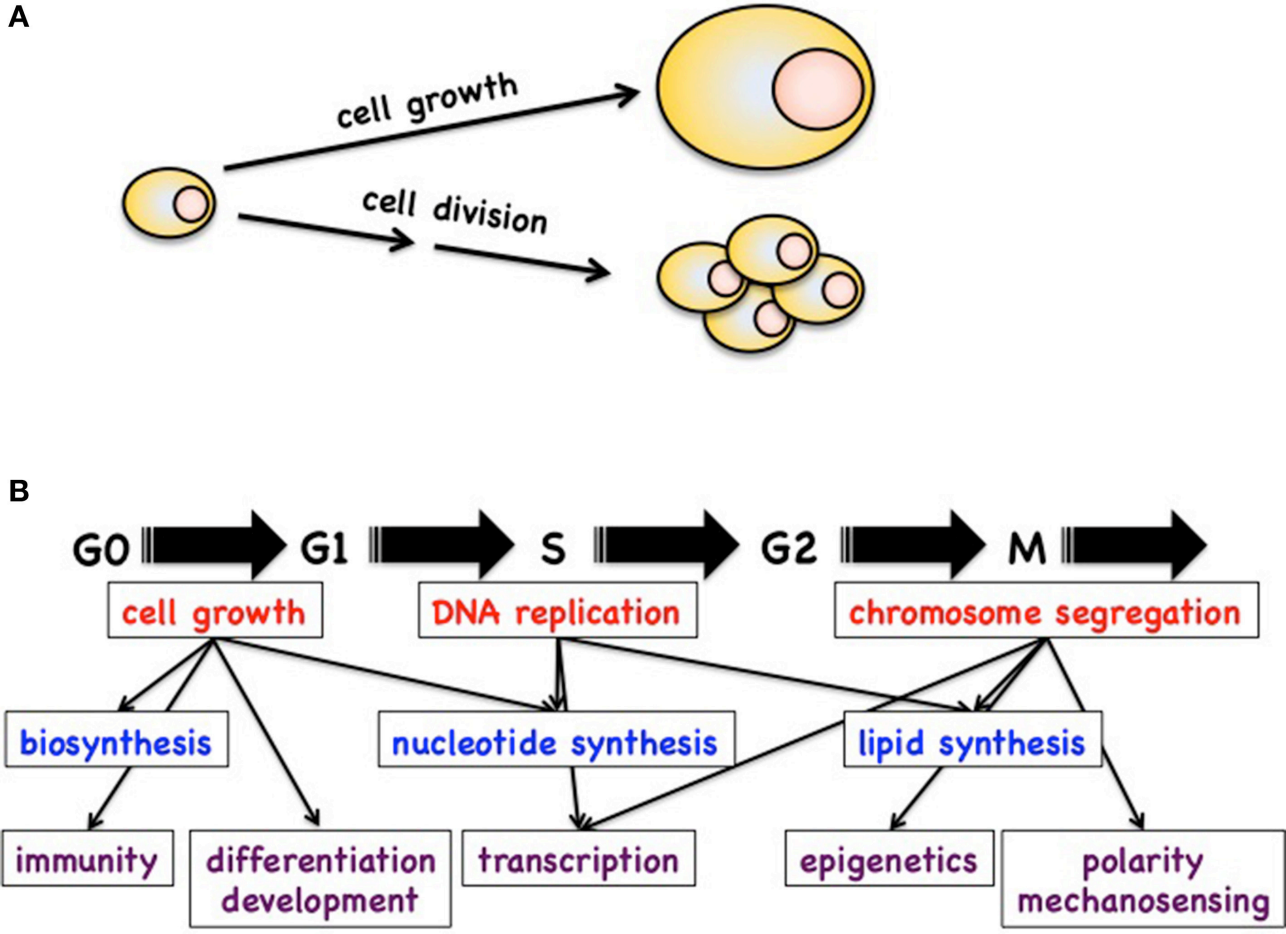
**(A)** Definition of cell growth and cell division. **(B)** Schematic of possible cross talk between the cell cycle machinery and processes like cell growth, transcription, epigenetics, biosynthesis, polarity, etc.

Cell growth refers to the increase in cell size (mass accumulation) while cell division describes the division of a mother cell into two daughter cells (1->2->4->8, etc.). Cell proliferation is the process of generating an increased number of cells through cell division. Both cell division and growth are tightly linked to the cell cycle and its regulation. For instance most of the cell growth in mammalian cells takes place during the G1 phase whereas in fission yeast it occurs mostly during the G2 phase (Mitchison, 2003; Navarro et al., 2012). Similarly, cell division has been studied extensively by investigating cell cycle regulation (Morgan, 2007). It requires prior duplication and separation of the complete genetic material of the mother cell (Gopinathan et al., 2011; Lim and Kaldis, 2013) and results in cytokinesis and the formation of two identical daughter cells. Given that cell growth and cell division are tightly connected and that half of the material of the mother cell is required each time to generate viable daughter cells, cell division cannot generally be achieved without proper cell growth. The existence of sensing mechanisms or a checkpoint of cell size was proposed quite some time ago. Despite this, the exact nature of the cell size checkpoint remains a subject of debate and its regulation elusive (Conlon and Raff, 1999). Nevertheless, there exist situations where cell division occurs in the absence of cell growth: for example amphibian eggs divide multiple times after fertilization to generate smaller cells with each division (Hörmanseder et al., 2013) and similar findings are also true for mammalian oocytes, where the initial divisions after fertilization are reductionist, which means that the size of the cells after division is approximately half of the mother cell due to lack of growth, which is also reflected by a short G1 phase (for a review see Palmer and Kaldis, 2016). Other examples include division of nuclei in the *Drosophila* embryonic syncytium, where 256 nuclei divide in the absence of growth of the oocyte (O'Farrell et al., 1989) and the final divisions of erythroid progenitors before the enucleate to become erythrocytes (Jayapal et al., 2010, 2015). In addition, under certain conditions stem cells can divide asymmetrically whereas the two daughter cells have different fates (Roubinet and Cabernard, 2014; Matsuzaki and Shitamukai, 2015; Chen et al., 2016).

## Cell division or the mitotic cell cycle

Cell cycle regulation has been studied extensively over the past decades and covered by many reviews and books (Morgan, 2007) but numerous aspects of the mitotic cell cycle remain elusive. Substantial gaps requiring further investigation exist to fully understand these mechanisms. These include for instance, how the origins of DNA replication are selected, how the spindle assembly checkpoint (SAC) is turned off once all chromosomes are bi-stably attached to microtubules, why the anaphase promoting complex/cyclosome (APC/C) is made up of numerous subunits and the functions of those subunits, how the timing of protein degradation is regulated during mitosis, and many more. The trend over the last 20 years has been to simplify scientific “stories” resulting in a whitewashing that can obscure details that make up the complexity of biological systems. One particular problem is the validity of generalizing *in vitro* mechanistic data from a particular cell line and extrapolating this to all cell types, tissues, and organisms. Therefore, as we progress, it is important to keep in mind the experimental context in which we study the processes of our interest.

In addition, our knowledge of the regulation of the meiotic cell cycle lags behind. There are obvious differences between mitosis and meiosis but meiosis also differs between females [ovary] and males [testis] (Clift and Schuh, 2013; Ohkura, 2015). These are major challenges to be uncovered in the future.

## Cell growth

Cell growth has been studied comprehensively in a variety of organisms and led to the identification of new regulatory pathways including mTOR, Myc, Hippo, and many others. The mTOR pathway senses multiple inputs and modulates the availability of energy and nutrients. The mTOR pathway is central for the regulation of cell growth (Laplante and Sabatini, 2012; Takahara and Maeda, 2013) as it regulates (and is also regulated) by growth factors, protein and lipid synthesis, autophagy, lysosome biogenesis, cell survival, cytoskeletal organization, and energy metabolism. The Hippo pathway is a kinase cascade that was originally identified in *Drosophila* and which regulates TEAD transcription factors that control cell proliferation, migration, and survival (Meng et al., 2016). The Hippo pathway receives its inputs from multiple cues including mechanobiology, stress signals, G-protein-coupled receptors, the cell cycle, and polarity (Meng et al., 2016). The transcription factor Myc regulates many genes involved in metabolism and cell growth (Stine et al., 2015). Cell growth is manifested itself in mass accumulation, which results in increased cell size. This has been intensively studied but the molecular determinants of cell size are still elusive (Ginzberg et al., 2015; Kiyomitsu, 2015; Schmoller and Skotheim, 2015; Amodeo and Skotheim, 2016). Since we have yet to completely understand the regulation of cell size (Lloyd, 2013), it is not surprising that the determinants of organ size are not known either (Hariharan, 2015; Penzo-Méndez and Stanger, 2015). Investigation of the molecular mechanisms controlling cell and organ size is definitely a grand challenge awaiting to be solved.

## Interplay of cell division with cell growth, biosynthesis, metabolism, immune response, epigenetics, mechanosensing, and others

Although the regulation of the cell cycle and cell growth is fairly well documented in the literature, we still do not fully understand how these processes are connected and regulate each other (Figure 1). Several fundamental observations have however indicated that these connections do exist and are important. The best example is that cells deprived of specific nutrients (preventing cell growth) cannot further progress through the cell cycle and thus cell division is blocked. On the other hand, cells that are arrested in the G1 phase can continue to grow without restrictions. In this context, it is obvious that cells progressing through the cell cycle require large amounts of energy, nucleotides, metabolites, and newly synthesized proteins and lipids. Nevertheless, in many cases we do not know how the cell cycle machinery communicates with the metabolic pathways to ensure that metabolites are sufficient at a given point of the cell cycle. Therefore, one of the grand challenges will be to define the cross talk between the cell cycle machinery and the metabolic pathways.

During cell proliferation the nutrient demands affect metabolism, whose alterations enable cells to meet biosynthetic requirements associated with cell growth and division. Therefore, the cross talk between cell growth, cell division, biosynthesis, metabolism, and various additional processes is crucial to maintain cellular homeostasis. Several points of contact are already known for the cross talk between the cell cycle machinery and metabolism. Among the best-known discoveries are the regulation of lipid synthesis by Cdk1 (Kurat et al., 2009; Miettinen et al., 2014), regulation of ribonucleotide reductase (Elledge et al., 1992; D'Angiolella et al., 2012; Guarino et al., 2014), regulation of glycolysis and mitochondria function (Lopez-Mejia and Fajas, 2015; Denechaud et al., 2016), and Cdk1 phosphorylating complex I (CI) subunits in the respiratory chain (Wang et al., 2014). It will be interesting to see these contact points expanded in the future and to confirm the detailed cross talk between the cell cycle machinery and metabolism.

The link between the cell cycle machinery and metabolism is not an isolated case and it is likely that there is also cross talk with other pathways like epigenetic regulators, mechanosensing, the immune system, and especially transcription. From these, the best understood aspect of regulation is the interplay of transcription, the cell cycle related enzymes, and protein degradation (Morgan, 2007). The accurate coordination of these processes is of great importance and the progress we have made in this scientific area over the last years is enormous. On the other hand, we know very little about the question of whether the immune system is regulated by the cell cycle machinery and if so, how this occurs (Rossi et al., 2006; Schmitz and Kracht, 2016). In addition, there are first indications that epigenetic regulators are controlled by cell cycle proteins (for example Chen et al., 2010) and surely mechanobiology will be soon linked to cell cycle regulators. Therefore, we are at the beginning of a new area to investigate the cross talk of the cell cycle machinery with other pathways and it will be interesting to see the development unfold in front of us.

## Goals of the cell growth and division section

We aim to be an open forum for studies that deal with cell cycle regulation, cell growth, cell division, and any other biological processes that are regulated by them. Interplay of two or more different pathways is an aspect of biological complexity that has not been extensively studied and we encourage submission of manuscripts in this area. In addition, we fully support the diversity of model systems used for our studies, which includes human cells, mice, worms, flies, yeast, zebrafish, frogs, sea urchin, etc. We hope to steer away our scientific curiosity from impact factors toward advancing the field step-by-step and letting history decide about the impact of our discoveries. We seek open discussions about controversial facts and observations. Therefore, opinion pieces, reviews, and commentaries are welcome as long as they do not ignore the rich data that has been already published.

## Author contributions

The author confirms being the sole contributor of this work and approved it for publication.

### Conflict of interest statement

The author declares that the research was conducted in the absence of any commercial or financial relationships that could be construed as a potential conflict of interest.

## References

[B1] Amodeo A. A. Skotheim J. M. (2016). Cell-size control. Cold Spring Harb. Perspect. Biol. 8:a019240. 10.1101/cshperspect.a019083 26254313 PMC4744813

[B2] Chen C. Fingerhut J. M. Yamashita Y. M. (2016). The ins(ide) and outs(ide) of asymmetric stem cell division. Curr. Opin. Cell Biol. 43, 1–6. 10.1016/j.ceb.2016.06.001 27318429 PMC5154912

[B3] Chen S. Bohrer L. R. Rai A. N. Pan Y. Gan L. Zhou X.. (2010). Cyclin-dependent kinases regulate epigenetic gene silencing through phosphorylation of EZH2. Nat. Cell Biol. 12, 1108–1114. 10.1038/ncb2116 20935635 PMC3292434

[B4] Clift D. Schuh M. (2013). Restarting life: fertilization and the transition from meiosis to mitosis. Nat. Rev. Mol. Cell Biol. 14, 549–562. 10.1038/nrm3643 23942453 PMC4021448

[B5] Conlon I. Raff M. (1999). Size control in animal development. Cell 96, 235–244. 10.1016/S0092-8674(00)80563-2 9988218

[B6] D'Angiolella V. Donato V. Forrester F. M. Jeong Y. T. Pellacani C. Kudo Y.. (2012). Cyclin F-mediated degradation of ribonucleotide reductase M2 controls genome integrity and DNA repair. Cell 149, 1023–1034. 10.1016/j.cell.2012.03.043 22632967 PMC3616325

[B7] Denechaud P. D. Lopez-Mejia I. C. Giralt A. Lai Q. Blanchet E. Delacuisine B.. (2016). E2F1 mediates sustained lipogenesis and contributes to hepatic steatosis. J. Clin. Invest. 126, 137–150. 10.1172/JCI81542 26619117 PMC4701565

[B8] Elledge S. J. Zhou Z. Allen J. B. (1992). Ribonucleotide reductase: regulation, regulation, regulation. Trends Biochem. Sci. 17, 119–123. 10.1016/0968-0004(92)90249-9 1412696

[B9] Ginzberg M. B. Kafri R. Kirschner M. (2015). Cell biology. On being the right (cell) size. Science 348:1245075. 10.1126/science.1245075 25977557 PMC4533982

[B10] Gopinathan L. Ratnacaram C. K. Kaldis P. (2011). Established and novel Cdk/cyclin complexes regulating the cell cycle and development. Results Probl. Cell Differ. 53, 365–389. 10.1007/978-3-642-19065-0_16 21630153

[B11] Guarino E. Salguero I. Kearsey S. E. (2014). Cellular regulation of ribonucleotide reductase in eukaryotes. Semin. Cell Dev. Biol. 30, 97–103. 10.1016/j.semcdb.2014.03.030 24704278

[B12] Hariharan I. K. (2015). Organ size control: lessons from *Drosophila*. Dev. Cell 34, 255–265. 10.1016/j.devcel.2015.07.012 26267393 PMC4547687

[B13] Hörmanseder E. Tischer T. Mayer T. U. (2013). Modulation of cell cycle control during oocyte-to-embryo transitions. EMBO J. 32, 2191–2203. 10.1038/emboj.2013.164 23892458 PMC3746200

[B14] Jayapal S. R. Lee K. L. Ji P. Kaldis P. Lim B. Lodish H. F. (2010). Down-regulation of Myc is essential for terminal erythroid maturation. J. Biol. Chem. 285, 40252–40265. 10.1074/jbc.M110.181073 20940306 PMC3001006

[B15] Jayapal S. R. Wang C. Q. Bisteau X. Caldez M. J. Lim S. Tergaonkar V.. (2015). Hematopoiesis specific loss of Cdk2 and Cdk4 results in increased erythrocyte size and delayed platelet recovery following stress. Haematologica 100, 431–438. 10.3324/haematol.2014.106468 25616574 PMC4380715

[B16] Kiyomitsu T. (2015). Mechanisms of daughter cell-size control during cell division. Trends Cell Biol. 25, 286–295. 10.1016/j.tcb.2014.12.003 25548067

[B17] Kurat C. F. Wolinski H. Petschnigg J. Kaluarachchi S. Andrews B. Natter K.. (2009). Cdk1/Cdc28-dependent activation of the major triacylglycerol lipase Tgl4 in yeast links lipolysis to cell-cycle progression. Mol. Cell 33, 53–63. 10.1016/j.molcel.2008.12.019 19150427

[B18] Laplante M. Sabatini D. M. (2012). mTOR signaling in growth control and disease. Cell 149, 274–293. 10.1016/j.cell.2012.03.017 22500797 PMC3331679

[B19] Lim S. Kaldis P. (2013). Cdks, cyclins and CKIs: roles beyond cell cycle regulation. Development 140, 3079–3093. 10.1242/dev.091744 23861057

[B20] Lloyd A. C. (2013). The regulation of cell size. Cell 154, 1194–1205. 10.1016/j.cell.2013.08.053 24034244

[B21] Lopez-Mejia I. C. Fajas L. (2015). Cell cycle regulation of mitochondrial function. Curr. Opin. Cell Biol. 33, 19–25. 10.1016/j.ceb.2014.10.006 25463842

[B22] Matsuzaki F. Shitamukai A. (2015). Cell division modes and cleavage planes of neural progenitors during mammalian cortical development. Cold Spring Harb. Perspect. Biol. 7:a015719. 10.1101/cshperspect.a015719 26330517 PMC4563714

[B23] Meng Z. Moroishi T. Guan K. L. (2016). Mechanisms of Hippo pathway regulation. Genes Dev. 30, 1–17. 10.1101/gad.274027.115 26728553 PMC4701972

[B24] Miettinen T. P. Pessa H. K. Caldez M. J. Fuhrer T. Diril M. K. Sauer U.. (2014). Identification of transcriptional and metabolic programs related to mammalian cell size. Curr. Biol. 24, 598–608. 10.1016/j.cub.2014.01.071 24613310 PMC3991852

[B25] Mitchison J. M. (2003). Growth during the cell cycle. Int. Rev. Cytol. 226, 165–258. 10.1016/S0074-7696(03)01004-0 12921238

[B26] Morgan D. O. (2007). The Cell Cycle: Principles of Control. London: New Science Press.

[B27] Navarro F. J. Weston L. Nurse P. (2012). Global control of cell growth in fission yeast and its coordination with the cell cycle. Curr. Opin. Cell Biol. 24, 833–837. 10.1016/j.ceb.2012.10.015 23182517

[B28] O'Farrell P. H. Edgar B. A. Lakich D. Lehner C. F. (1989). Directing cell division during development. Science 246, 635–640. 2683080 10.1126/science.2683080

[B29] Ohkura H. (2015). Meiosis: an overview of key differences from mitosis. Cold Spring Harb. Perspect. Biol. 7:a015859. 10.1101/cshperspect.a015859 25605710 PMC4448623

[B30] Palmer N. Kaldis P. (2016). Regulation of the embryonic cell cycle during mammalian preimplantation development. Curr. Top. Dev. Biol. 120, 1–53. 10.1016/bs.ctdb.2016.05.001 27475848

[B31] Penzo-Méndez A. I. Stanger B. Z. (2015). Organ-size regulation in mammals. Cold Spring Harb. Perspect. Biol. 7, a019240. 10.1101/cshperspect.a019240 26187729 PMC4563708

[B32] Rossi A. G. Sawatzky D. A. Walker A. Ward C. Sheldrake T. A. Riley N. A.. (2006). Cyclin-dependent kinase inhibitors enhance the resolution of inflammation by promoting inflammatory cell apoptosis. Nat. Med. 12, 1056–1064. 10.1038/nm1468 16951685

[B33] Roubinet C. Cabernard C. (2014). Control of asymmetric cell division. Curr. Opin. Cell Biol. 31, 84–91. 10.1016/j.ceb.2014.09.005 25264944

[B34] Schmitz M. L. Kracht M. (2016). Cyclin-dependent kinases as coregulators of inflammatory gene expression. Trends Pharmacol. Sci. 37, 101–113. 10.1016/j.tips.2015.10.004 26719217

[B35] Schmoller K. M. Skotheim J. M. (2015). The biosynthetic basis of cell size control. Trends Cell Biol. 25, 793–802. 10.1016/j.tcb.2015.10.006 26573465 PMC6773270

[B36] Stine Z. E. Walton Z. E. Altman B. J. Hsieh A. L. Dang C. V. (2015). MYC, metabolism, and cancer. Cancer Discov. 5, 1024–1039. 10.1158/2159-8290.CD-15-0507 26382145 PMC4592441

[B37] Takahara T. Maeda T. (2013). Evolutionarily conserved regulation of TOR signalling. J. Biochem. 154, 1–10. 10.1093/jb/mvt047 23698095

[B38] Wang Z. Fan M. Candas D. Zhang T. Q. Qin L. Eldridge A.. (2014). Cyclin B1/Cdk1 coordinates mitochondrial respiration for cell-cycle G2/M progression. Dev. Cell 29, 217–232. 10.1016/j.devcel.2014.03.012 24746669 PMC4156313

